# Retraction Note: Unveiling the potential of machine learning approaches in predicting the emergence of stroke at its onset: a predicting framework

**DOI:** 10.1038/s41598-026-54512-1

**Published:** 2026-05-26

**Authors:** Sheela Lavanya J M, Subbulakshmi P

**Affiliations:** School of Computer Science and Engineering, Vellore Institute of Technology, Chennai, India

Retraction Note to: *Scientific Reports* 10.1038/s41598-024-70354-1, published online 29 August 2024

The Editors have retracted this Article.

After publication, concerns were raised regarding the dataset used in this study [1]. The Authors relied on a publicly available dataset, the Stroke Prediction Dataset, whose provenance and validity cannot be confirmed. As this dataset was used to train and assess the machine-learning models examined in this Article, the Editors no longer have confidence in the reliability of the results and conclusions of this Article.

Sheela Lavanya J M and Subbulakshmi P disagree with this retraction.
